# Impact of rheumatoid factors on the function of therapeutic monoclonals specific for PD-1/PD-L1

**DOI:** 10.1007/s00262-025-04078-0

**Published:** 2025-05-24

**Authors:** Barry D. Hock, Liping Goddard, Lachlan J. Dobson, Sean A. MacPherson, John L. O’Donnell, Judith L. McKenzie, Alexander D. McLellan

**Affiliations:** 1 Haematology Research Group, Department of Pathology and Biomedical Science, University of Otago, Christchurch, New Zealand; 2 Department of Microbiology and Immunology, University of Otago, Dunedin, New Zealand; 3 Haematology Department, Christchurch Hospital, Christchurch, New Zealand; 4 Immunology Department of Canterbury Health Laboratories, Christchurch Hospital, Christchurch, New Zealand

**Keywords:** Rheumatoid factor, PD-1, PD-L1, Antibody, Fc receptor

## Abstract

The efficacy of blocking antibodies against programmed death-1 (PD-1) and its ligand (PD-L1) is modulated by signalling through their Fc regions. The Fc region of anti-PD-1/PD-L1 antibodies, when cell-bound, represents a potential target for recognition by circulating rheumatoid factor (RF) autoantibodies. The resultant cell-associated immune complex may then provide different Fc signals to that of the PD-1/PD-L1 antibodies alone. However, little is known regarding the interaction of RF and therapeutic PD-1/PD-L1 antibodies. We report that PD-1 (pembrolizumab, nivolumab) and PD-L1 (avelumab) antibodies, when bound to their cellular targets, are recognised by both IgM-RF and IgA-RF components of RF^+^ patient serum. We further demonstrate that the presence of RF provides PD-1 antibodies with the ability to induce complement-dependent cytotoxicity (CDC) of a PD-1^+^ target cell line in the presence of human complement. Although RF provided avelumab with the ability to induce CDC in assays using rabbit complement, no CDC was observed in the presence of human complement. The presence of RF did not modulate the level of Fc receptor-triggered cellular cytotoxicity or neutrophil activation that was induced by PD-1/PD-L1 antibodies alone. This study demonstrates that RF has the potential to modulate the Fc-associated signals generated following binding of PD-1/PD-L1 antibodies. The impact of RF on their efficacy therefore merits further investigation.

**Supplementary Information:**

The online version contains supplementary material available at 10.1007/s00262-025-04078-0.

## Introduction

Therapeutic antibodies that bind PD-1 or PD-L1, and thereby block delivery of inhibitory signals through the PD-1/PD-L1 axis, have revolutionised the treatment of malignancies such as melanoma [1]. The functional effects of therapeutic antibodies result from not only antigen binding, but also the subsequent, isotype-dependent, signals delivered by their Fc regions [2–4]. Fc-mediated signalling has been shown to modulate the anti-tumour activity of anti-PD-1/PD-L1 mAbs [3, 5–10] and therefore understanding the Fc-mediated signals delivered following binding of these antibodies is critical to optimising their use.

The PD-1-specific therapeutic antibodies pembrolizumab and nivolumab were designed as IgG_4_ in order to avoid triggering Fc-mediated antibody-directed cellular cytotoxicity (ADCC) and complement-dependent cytotoxicity (CDC) [2, 4]. However, these IgG_4_ antibodies can still provide some Fc receptor (FcR) signalling [11], which, in some settings, can abrogate the benefits of PD-1 blockade [6, 8]. Therefore, Fc null variants of PD-1 antibodies are currently thought to have the most clinical utility [3]. It is presently unclear whether Fc function enhances or inhibits the therapeutic activity of PD-L1 targeted antibodies [3, 4]. To date, efforts to optimise Fc signalling via PD-1/PD-L1 antibodies have been based on the premise that only the target-bound mAb provides Fc signalling [12]. However, if these bound therapeutics are, themselves, recognised by antibodies of differing isotypes, there is potential for the resulting surface bound immune complex (IC) to trigger an additional range of Fc-mediated effects. We have previously demonstrated that the binding of in vivo generated anti-drug antibodies and anti-hinge antibodies to pembrolizumab can modulate the Fc signalling relative to pembrolizumab alone [13, 14]. Another group of in vivo generated antibodies with the potential to recognise therapeutic monoclonals are rheumatoid factors (RFs).

RFs are autoantibodies that do not recognise native IgG but recognise epitopes on the Fc region of IgG that are exposed following conformational changes induced by antigen binding or IgG aggregation/denaturation [15]. They are comprised of multiple isotypes, predominantly IgM/ IgA and recognise a range of epitopes, with individuals varying considerably with regard to the isotypes and epitope specificity of their RF repertoire [16, 17]. RFs are not only present in most patients with rheumatoid arthritis, but also in subsets of patients with other autoimmune diseases, B cell malignancies or infections [18]. Although low in the general population, RF prevalence increases with age. Studies on the functional activity of RF have been limited to date. The incorporation of RF into solid-phase or soluble IC has been predominantly reported to increase complement activation and variably reported as either inhibiting or increasing FcR binding [16, 19–22]. Studies on the potential impact of RF on therapeutic antibody function have been restricted to a report that RF inhibited the CDC resulting from exposure of a lymphoma cell line to the CD20 mAb rituximab [23]. The binding of RF to cell-bound PD-1/PD-L1 monoclonals has the potential to result in the formation of an IC on the cell surface comprised of multiple isotypes. The IC may then potentially induce a different patterns of FcR and complement activation compared to the monoclonal alone. These alterations in Fc-mediated signalling may in turn modulate the nature and/or magnitude of clinical responses observed following treatment with PD-1/PD-L1 antibody. Studies to date have reported conflicting results as to whether RF positivity was associated with of good or poor prognosis in cancer patients receiving such therapies [24–27]. A single in vitro study reported that RF does not inhibit the binding of nivolumab to PD-1 [25], but the interaction between PD-1/PD-L1 antibodies and RF has otherwise received little study.

In this study, we have investigated the binding of RF to PD-1/PD-L1 antibodies in current clinical usage and analysed the functional outcomes of their interaction.

## Materials and methods

### Cell and serum preparation

Blood was collected with written consent from (i) normal donors (ND) and (ii) patients attending rheumatology clinics who had been diagnosed with rheumatoid arthritis (RA) as defined by the American College of Rheumatology 2010 classification criteria. Samples from RA patients were analysed for the presence of IgM-RF as described below and then subdivided into those lacking detectable RF (RF^Neg^) or those containing RF (RF^Pos^). Ethical approval was obtained from Health and Disability Ethics Committees, New Zealand.

Serum was obtained by incubation (2 h/RT) then centrifugation (1200×g/20 min) of blood and then stored frozen.

Serum for use as a complement source was prepared from rabbit or human whole blood that was allowed to clot (30 min, RT) prior to centrifugation (500xg, 10 min). Recovered serum was stored in aliquots at − 80 °C and thawed immediately prior to use.

The R10 media used in cell-based experiments was RPMI (Sigma, St Louis, MO) supplemented with 10% heat inactivated foetal calf serum (FCS, Invitrogen, Auckland, New Zealand)] glutamine and penicillin/streptomycin unless otherwise indicated.

The B cell lines Raji and Ramos were obtained from ATCC (Manassas, VA, USA) and maintained in R10 media. PD-1^+^ Ramos and PD-L1^+^ Ramos were generated as follows. Full length PD-1 and PD-L1 cDNA sequences were synthesised by Twist Biosciences then restriction cloned using SfiI into Sleeping Beauty vectors under the control of the EF-1 promoter [28]. PD-1 or PDL-1 vectors were then transposed into Ramos using a neon electroporator [29]. Following two weeks of antibiotic selection, PD-1 or PDL-1 expression was confirmed by flow cytometry.

A natural killer cell line (GFP-CD16-NK-92) that had been transduced to express GFP and high-affinity CD16 (V/V) was obtained from the ATCC and maintained in R10 media supplemented with horse serum, sodium pyruvate, non-essential amino acids and 100 U/ml IL-2 as described [30]. Granulocytes were prepared by centrifugation over Ficoll/Paque in combination with NH_4_Cl lysis as described [14].

### Therapeutic monoclonals

Stocks of therapeutic monoclonals were obtained from injection vials of rituximab (MabThera, Roche), nivolumab (OPDIVO, Bristol-Myers Squibb), pembrolizumab (Keytruda, Merck) and natalizumab (Tysabri, Biogen). Avelumab was produced in house using ExpiCHO. In brief, codon-optimised Avelumab heavy and light chain sequences were synthesised by Twist biosciences and then restriction cloned into pcDNA3.1 plasmid. ExpiCHO-S cells (A29127, Thermo Fisher) were transfected with a 1:1 weight ratio of heavy and light chain plasmids using the Expifectamine CHO transfection kit (Gibco). Avelumab was purified from the supernatant of cultured transfectants using protein A. F(ab’)_2_ fragments of pembrolizumab and rituximab were generated and purity confirmed as described previously [14].

### ELISA’s for IgM-RF and IgA-RF

For detection of RF binding to solid-phase antibody ELISA plates (Costar) were coated (16 h, 4 °C) with 2 µg/ml F(ab’)_2_/IgG, washed (0.1% Tween 20/PBS), blocked (1 h, 1% milk powder/PBS (MP)) then incubated (90 min/37 °C) with serum samples (0.2% in diluent (1% MP, 0.1% Tween 20), washed and incubated (1 h/37 °C) with either (i) goat anti-human IgA biotin [Invitrogen] diluted in 1% MP, 5% goat serum, washed. Then, incubated (30 min/37 °C) with streptavidin–horseradish peroxidase (HRP) enzyme or (ii) HRP-conjugated mouse anti-human IgM (Invitrogen) diluted in 0.1% BSA, 0.1% Tween-PBS. In specificity experiments, diluted serum was pre-incubated (30 min) with either nil, monoclonal or heat-aggregated monoclonal (20 mg/ml heated at 63 °C, 25 min) at 200 ug/ml prior to analysis. For detection of RF binding to PD-1 bound, antibody ELISA plates were coated (16 h/4 °C) with 2 ug/ml recombinant His-PD-1(R&D systems), washed, blocked (MP), incubated (1 h/37 °C) with mAb (2 ug/ml in MP), washed then processed as described above. Bound HRP was detected using TMB + substrate (Dako), stopped with 3 mol/L H_2_SO_4_ and absorbance read at 450 nm.

### Detection of RF binding by flow cytometry

IgA-RF was detected by staining PD-1^+^-Ramos. Cells were incubated (30 min/RT) with 0 or 10 ug/ml monoclonal in the presence of 4% ND or RF^+^ sera. Following washing (PBS), cells were incubated (30 min/RT) with Fitc-goat anti-human IgA (Invitrogen) then washed and analysed. IgM-RF was detected by staining PD-1^+^-Jurkat as above and bound RF detected using Fitc-sheep anti-human IgM mu chain (Chemi-Con). Flow cytometric analysis was performed on a Beckman Coulter Cytoflex S flow cytometer, and results expressed as mean fluorescence intensity (MFI).

### Complement-dependent cytotoxicity (CDC) assay

Target cells (1 × 10^5^) in 20 µl C´media (1:1 AIM V: HBSS (Gibco)) were added to wells of a 96 well U bottom plate together with 20 µl of C´media supplemented with either nil, antibody or F(ab’)_2_ fragments (20 µg/ml). Following incubation (10 min/RT), 10 µl of ND or RF + sera (20% in C´media) was added, further incubated (30 min/RT) then 100 µl of C´ serum (16% rabbit serum or human serum in HBSS) was added then incubated (2 h /37 °C) prior to addition of propidium iodide (PI) and determination of the proportion of PI^+^ non-viable cells by flow cytometry. In specificity experiments, the C´ serum was supplemented with 30 mM EDTA as indicated. Each ND/RF^+^ serum sample was incubated in both the presence and absence of antibody/ F(ab’)_2_ and the difference between the treatments provided the measure of specific lysis.

### Statistics

Statistical comparisons were performed as indicated in legends. Asterisks indicate significance ^*^P < 0.05; ^**^P < 0.01; ^***^P < 0.001; ^****^P < 0.0001. All analysis/graphing was performed using GraphPad Prism version 10.0.0 for Windows (GraphPad Software, La Jolla, California USA).

### NK assay

Target cells were fluorescently labelled with CellTrace Yellow (CTY, Invitrogen) immediately prior to use according to manufacturer’s instructions. The assay was then performed in C´media. CTY^+^ targets (20 µl at 2.5 × 10^6^/ml) were incubated (10 min/RT) with 20 µl mAb (20 µg/ml) pre-addition of test sera (10 µl at 20%v/v). Following incubation (30 min/RT), NK-92 (100 µl at 2 × 10^6^/ml) was added and cultures incubated 3.5 h prior to addition of 50 µl SYTOX BLUE diluted in 20 mM EDTA/PBS and analysis by flow cytometry. Target cytotoxicity was defined as % SYTOXBLUE^+^.

### Granulocyte activation assay

Granulocytes (3 × 10^6^/ml in R10) were incubated (15 min/37 °C) with the reactive oxygen species (ROS) indicator dihydrorhodamine 123 (DHR, Invitrogen, 5 uM), and then following adjustment of DHR concentration to 10 µM, 50 ul was added to a coated ELISA plate.

ELISA plates were coated (2 h, 37 °C) with antibody (10 µg/ml), washed and blocked. Wells were them pre-incubated (30 min) with 50 µl of ND or RF sera (0.5% in R10) pre-addition of 50 µl DHR^+^ neutrophils. Following incubation (1 h/37 °C), the MFI of green fluorescent rhodamine 123 was determined by flow cytometry.

## Results

### Reactivity of RF

**Fig. 1:**
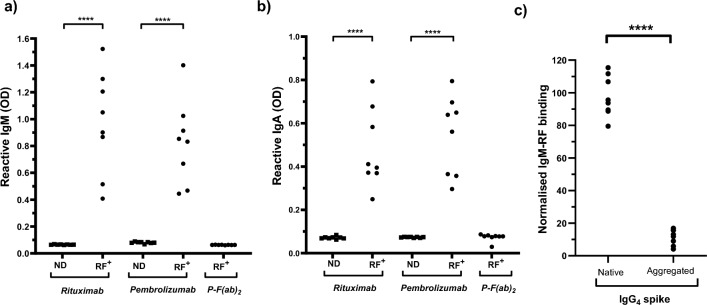
Specificity of RF^+^ serum reactivity with monoclonals. Serum from normal donors (NDs) and patients with reported RF (RF^+^) were analysed with respect to reactivity with therapeutic monoclonals. **a**, **b** ND and RF^+^ sera reactivity with either rituximab, pembrolizumab or the F(ab)_2_ fragment of pembrolizumab (p-F(ab)_2_) was analysed by ELISA specific for either **a** IgM or **b** IgA antibodies. Data are shown as a scatter plot of OD for each sample. Asterisks indicate significant differences between indicated ND and RF data following analysis by unpaired t-test **c** The specificity of RF reactivity was analysed by spiking each RF^+^ sample with either nil or nivolumab that was in its native form or heat aggregated. The level of IgM antibodies reactive with solid-phase pembrolizumab in each sample was then determined by ELISA and normalised relative to the level in the corresponding nil-treated sample (defined as 100%). Data are shown as a scatter plot of normalised IgM-RF binding for all RF^+^ samples. Asterisks indicate significant differences following paired t-test

Sera from arthritis patients that had reactivity with solid-phase IgG_1_ by ELISA (n = 8) were defined as RF^+^ and characterised further. All RF^+^ sera contained both IgM-RF and IgA-RF reactive with immobilised forms of both IgG_1_ rituximab and IgG_4_ pembrolizumab (Fig. 1a,b). In contrast, no reactivity with F(ab)_2_-pembrolizumab was observed, thereby confirming RF specificity for Fc epitopes. A feature of RF is that they only recognise IgG that has undergone a conformational change induced by antigen binding, denaturation or immobilisation [15]. Inhibition experiments were therefore performed using IgG_4_ nivolumab to inhibit RF binding to immobilised pembrolizumab (Fig. 1c). The binding of all RF^+^ sera was strongly inhibited by the presence of heat denatured nivolumab but not by native nivolumab.

**Fig. 2:**
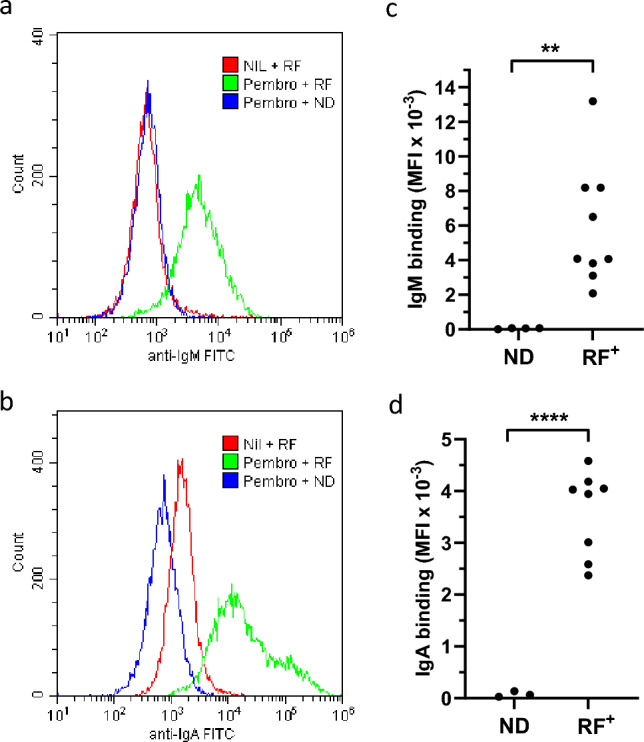
Reactivity of RF with pembrolizumab bound to cellular PD-1. Flow cytometric analysis of anti-IgM and anti-IgA staining of PD-1^+^ cell lines labelled with either nil or pembrolizumab in combination with ND or RF^+^ sera. **a**,**b** Representative histograms of staining observed using **a** Fitc-anti-IgM or **b** Fitc–anti-IgA to detect antibodies following incubation with the indicated combinations of pembrolizumab and ND/RF^+^ sera. **c**,**d** Scatter plot of specific MFI observed (pembrolizumab with sera—sera alone) for each individual serum following **c** anti-IgM or **d** anti-IgA detection. Asterisks indicate significant differences following unpaired t-test

The ability of RF to recognise pembrolizumab that was antigen-bound rather than immobilised was also analysed. In an ELISA, utilising solid-phase PD-1 neither IgM-RF nor IgA-RF was reactive with PD-1 alone but, unlike ND sera, was reactive with pembrolizumab bound to PD-1 (Supplementary Fig. 1). In addition, the binding of RF to a PD-1 expressing cell line was analysed by flow cytometry (Fig. 2). IgM-RF was unreactive and IgA-RF only weakly reactive with non-labelled cells. However, in contrast to ND sera, they were strongly reactive with PD-1^+^ cell lines that were pre-labelled with pembrolizumab. IgM-RF was similarly reactive with cell lines pre-labelled with nivolumab (Supplementary Fig. 2).

### Effect of RF on CDC induced by therapeutic antibodies

**Fig. 3:**
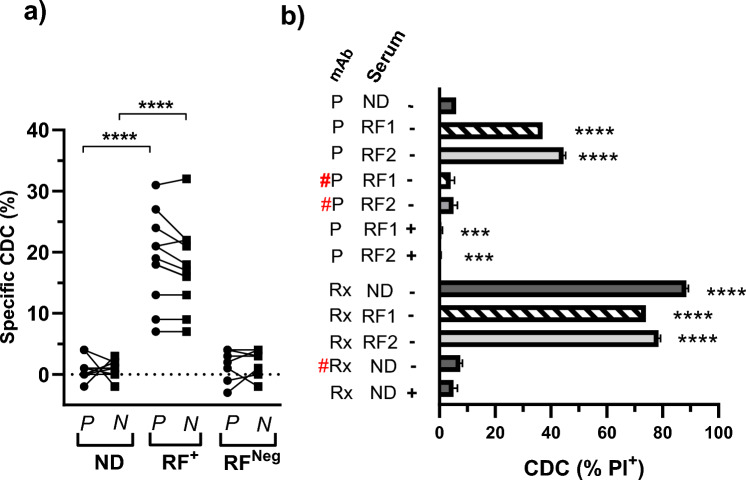
Effect of RF on CDC induced by PD-1 mAb. PD-1^+^ Ramos was incubated with combinations of ND/RF^+^ sera and monoclonal then exposed to human complement. **a** Scatter plot of the specific CDC observed using pembrolizumab (P) or nivolumab (N) in combination with either ND (n = 8), RF^+^ arthritis (n = 8) and RF^Neg^ arthritis (n = 7) sera. The specific CDC for each individual serum was calculated as the % CDC with mAb—CDC without mAb. Asterisks indicate significant differences between indicated groups following unpaired t-test. **b** Bar graph of the CDC observed using a mAb (pembrolizumab (P), rituximab (Rx) or the respective F(ab)_2_ fragments (#) in combination with the indicated sera (ND or RF^+^(RF1& RF2)). Complement was supplemented with (+) EDTA as indicated. Data are shown as % PI^+^ (mean ± SEM) of replicates from a representative experiment of two performed. Asterisks indicate a significant difference from levels in corresponding ND-treated cells following analysis by ANOVA in combination with Holm-Sidak multiple comparisons test

The ability of RF to induce CDC was first analysed using human serum as a complement source. In the assay, targets were mixed with test sera (ND, RF^+^) ± mAb then incubated with excess complement serum pre-analysis. The final complement serum: Test serum ratio was 7.6:1. Specific CDC was defined as the difference between RF + mAb and RF alone thereby controlling for any cytotoxic effects of the test serum alone. Analysis using PD-1^+^ Ramos as a target and utilising ND sera in combination with pembrolizumab or nivolumab detected only low levels of specific CDC (< 5%) (Fig. 3a). However, in the presence of RF^+^ sera, significantly higher levels of specific CDC were observed. In contrast, low levels of CDC were observed in arthritis patients who were RF^Neg^.

The specificity of pembrolizumab CDC was compared to that observed using rituximab, which is a well characterised initiator of CDC (Fig. 3b). Rituximab-induced high levels of CDC in the presence of both ND sera and RF^+^ sera, whereas pembrolizumab induced CDC only in the presence of RF^+^ sera. No CDC was observed using either F(ab)_2_-rituximab + ND sera or F(ab)_2_-pembrolizumab + RF sera, thereby confirming the requirement for a Fc region in both settings. As IgG_4_ are, by virtue of their structure, extremely poor activators of CDC [2, 31], the requirement for pembrolizumab to have a Fc region provides strong evidence that it is the Fc binding RF antibodies within the RF^+^ serum that induce complement activation. The CDC induced by both rituximab and the pembrolizumab/RF^+^ sera combination was also inhibited by EDTA confirming the involvement of the complement system.

The possibility that the difference between ND and RF^+^ sera may reflect differences in their intrinsic complement activity was investigated. No CDC was observed using pembrolizumab in combination with ND sera irrespective of whether it had full or nil complement activity (Supplementary Fig. 3). Furthermore, similarly low levels of complement activity were detected in ND and RF^+^ samples (Supplementary Fig. 4). The difference between ND and RF^+^ sera was still observed in CDC assays where test serum was washed away pre-addition of complement serum (Supplementary Fig. 5). This confirms that RF serum has its effect on CDC prior to actual complement activation.

It has been reported that RF can markedly inhibit rituximab-induced CDC of the B cell lymphoma cell line Daudi [23]. However, our analysis of rituximab-induced CDC of the B cell lines Raji and Ramos detected only small changes in CDC as a result of RF addition (Supplementary Fig. 6).

**Fig. 4:**
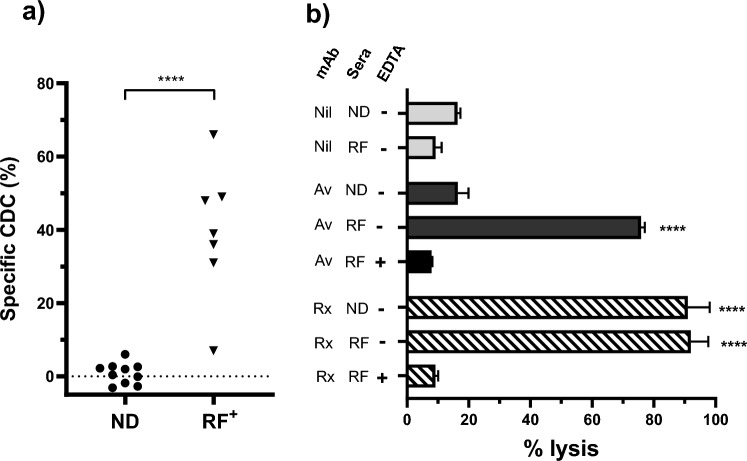
Effect of RF on CDC induced by PD-L1 mAb. PD-L1 ^+^ Ramos was incubated with combinations of ND/RF^+^  sera and monoclonal then exposed to rabbit complement. **a** Scatter plot of the specific CDC observed using avelumab plus either ND (n = 10) or RF^+^ (n = 7) sera. Specific CDC for each serum was calculated as the % CDC with—CDC without mAb. Asterisks indicate significant differences following unpaired t-test. **b** Bar graph of the CDC observed using a mAb (avelumab (Av) or rituximab (Rx)) in combination with ND or RF^+^ serum. Rabbit complement was supplemented with EDTA as indicated. Data are shown as % PI^+^ (mean ± SEM) of replicates from a representative experiment of two performed. Asterisks indicate a significant difference from levels in nil-treated cells following analysis by ANOVA in combination with Holm-Sidak multiple comparisons test

The ability of RF^+^ sera to modulate CDC induced by the PD-L1 monoclonal avelumab was also analysed using human complement and PD-L1^+^  Ramos as a target. Although strong CDC was induced using rituximab little specific CDC was observed using avelumab in combination with either ND or RF^+^ sera (data not shown). The CDC-inducing activity of avelumab ± RF was then analysed using rabbit sera as a complement source, as it’s less affected by human complement regulatory proteins, and is triggered more easily by some human antibody classes [32–35]. Preliminary experiments using rituximab + Ramos confirmed that rabbit complement induced higher levels of CDC than human complement (Supplementary Fig. 7). Using rabbit complement high levels of specific CDC were observed with 6/7 RF^+^sera when added to PD-L1^+^ Ramos in combination with avelumab (Fig. 4a). In contrast, little specific CDC was induced following addition of ND sera to Ramos + avelumab. The specificity of the observed CDC was compared to that observed using rituximab (Fig. 4b). Rituximab induced similarly high levels of CDC in the presence of both ND sera and RF^+^ sera. In contrast, avelumab induced CDC only in the presence of RF^+^ sera. The induction of CDC subsequent to both avelumab and rituximab binding was inhibited by the addition of EDTA to the complement source confirming the involvement of the complement system.

### Effect of RF on ADCC

**Fig. 5:**
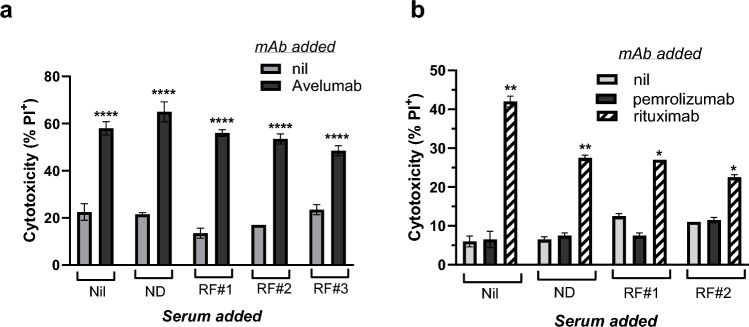
Effect of RF on ADCC. Cytotoxicity was assessed in a 4 h killing assay using NK-92 as the effector and either **a** PD-L1^+^ Ramos or **b** PD-1^+^ Ramos as the target at a 10:1 effector to target ratio. Cultures with additionally supplemented with the indicated combinations of monoclonal and either ND or RF^+^ sera. Data are shown as % specific lysis and are from a representative experiment of 2 performed. For each serum type, asterisks indicate antibody containing cultures significantly different to the corresponding no antibody cultures. All statistical analysis was by 2 way ANOVA in combination with Holm-Sidak multiple comparison test

ADCC was analysed using an NK cell line (NK-92) as the effector and Ramos as the target (Fig. 5). In cultures with PD-L1^+^ Ramos, only low levels of ADCC were observed in the absence of antibody whereas addition of avelumab strongly induced ADCC (Fig. 5a). The presence of either ND or RF^+^ sera did not modulate ADCC. In cultures containing PD-1^+^ Ramos, no ADCC was observed in either the absence or presence of pembrolizumab (Fig. 5b). The addition of ND or RF^+^ sera did not increase ADCC. In contrast, the addition of rituximab strongly induced ADCC. The addition of both ND and RF^+^ sera significantly (*p* < 0.001) inhibited the level of rituximab-induced ADCC, which is consistent with previous studies that utilised rituximab in combination with ND serum [36]

### Effect of antibody complexes on granulocyte activation

**Fig. 6:**
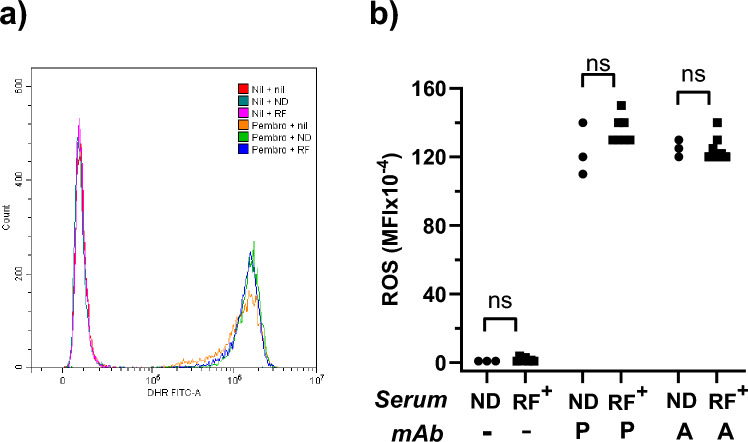
Effect of RF on ROS production by granulocytes. Granulocytes loaded with ROS indicator (DHR) were added to wells that were pre-coated with either nil, pembrolizumab (P) or avelumab (A) and additionally contained media supplemented with either nil, ND sera or RF^+^ sera. Plates were then incubated for 1 h prior to flow cytometric analysis of rhodamine 123 staining. **a** Representative histograms of rhodamine 123 staining observed following culture under the indicated conditions. Data are from a single experiment of 3 performed. **b** Scatter plot of the MFI (× 10^–4^) of rhodamine 123 staining observed in the presence of each individual ND sera (n = 3) or RF^+^ sera (n = 6) when added to nil or pembrolizumab-coated wells. Data are from a representative experiment of three performed. For each antibody type, ND and RF^+^ were compared by unpaired t-test and non-significance (ns) indicated. Granulocyte purity was > 90% in all preparations based on flow cytometric analysis of both scatter and CD66b expression

Granulocytes generate ROS in response to engagement of their FcR, in particular CD32, with IgG complexes [37]. The impact of RF on IgG complex induced ROS generation was therefore assessed (Fig. 6). Granulocytes incubated in the absence of solid-phase IgG had little detectable ROS generation, while the presence of either solid-phase pembrolizumab or avelumab strongly induced ROS production. Similar levels of ROS generation were observed in the presence of ND or RF^+^ sera.

## Discussion

Despite the likely presence of RF in populations receiving PD-1/PD-L1 antibodies, their potential impact on treatment efficacy is largely unknown.

In the current study, we analysed a set of RF^+^ sera and demonstrated that all contained IgM-RF and IgA-RF that recognise IgG_4_ anti-PD-1 antibodies carrying the S228P Fc mutation. Importantly, this reactivity was observed with both solid-phase and PD-1 bound antibody.

Pembrolizumab and nivolumab were engineered as IgG_4_ to avoid triggering CDC or ADCC. However, the subsequent binding of RF, particularly IgM-RF, has the potential to result in CDC. The current study, demonstrates that RF in combination with pembrolizumab/nivolumab, but not alone, specifically induces CDC of PD-1^+^ cells. This is the first demonstration of RF-inducing CDC of antibody labelled cells as previous studies analysed complement deposition on solid-phase antibodies [16, 19, 22]. Effective antibody induced CDC not only requires binding of complement fixing isotypes but is also critically dependent on factors such as antigen density, antigen movement following antibody binding and epitope location relative to the cell surface [38]. Therefore, these results demonstrate that the RF repertoire can, at least in the context of pembrolizumab/nivolumab binding, fulfil the multiple requirements for inducing CDC and thereby, potentially deplete PD-1^+^ cells.

Although PD-1^+^ T cells have been regarded as the primary target of PD-1 antibodies, it has become increasingly clear that PD-1 can be expressed by tumour cells as well as a range of immune cells [39]. Therefore, the clinical impact of RF will depend on whether the tumour cells or immune cells have the combination of PD-1 and complement regulatory protein expression that makes them CDC susceptible [38]. Studies on the association between RF positivity and outcomes in patients receiving PD-1 antibodies have provided conflicting results with two reporting increased immune-related events and improved survival [24, 26], while Ugolini et al. [25] reported an association with poor survival outcomes. A further study reported that patients with rheumatoid arthritis had similar survival to control although RF levels, which can be decreased by arthritis treatments, were not analysed [27]. These differences in findings suggest that the therapeutic impact of RF-driven CDC may vary depending on the tumour microenvironment and larger studies are required to address this.

Avelumab either alone, or in combination with the same set of RF sera, did not induce CDC suggesting that the antigen/epitope arrangement was less conducive to activation of human complement. The observation that CDC could be observed with avelumab + RF when rabbit complement was utilised supports this as rabbit complement is more readily activated in a human target/antibody setting [32–35]. This finding does however confirm that RF does bind to avelumab at a level that can be functionally active. This may allow human complement activation in a setting where target cells express higher levels of PD-L1 and/or lower levels of complement inhibitors.

Although RF bind primarily to Fc regions distinct to those involved in C1q and FcR binding [40, 41], it has been reported that RF inhibited rituximab-induced CDC of the B cell lymphoma line Daudi [23]. However, in the current study, RF did not inhibit rituximab-induced CDC of the B cell lymphoma lines Ramos and Raji. This difference may reflect the fact that Daudi, unlike Ramos and Raji, co-expresses BCR and CD32b which are known to modulate CD20 antibody induced CDC [42, 43].

It has also been reported that the binding of recombinant CD32 to immobilized IgG can be partially inhibited by at least some monoclonal RF [16, 21]. In the current study, the presence of RF modulated neither ADCC induced by CD16^+^ NK cells nor ROS generation by neutrophils in response to immobilised IgG, which is predominantly CD32 mediated [37]. This may reflect the use, in this study, of cells expressing FcR to detect functional outcomes of IgG binding. Notably, previous studies analysing FcR-stimulated macrophages found RF boosted rather than inhibited the responses [19, 20].

The focus of the current study was evaluating the impact of RF on functional activities such as CDC, PD-1-PD-L1 engagement and Fc gamma receptor (FcγR)-mediated signalling. However, the binding of IgA-RF and IgM-RF may also potentially induce signalling via IgA (FcαR) and IgM (FcµR) receptors and therefore provide additional immuno-modulatory signals in response to RF engagement.

RF can recognise multiple epitopes and it has been reported that RF repertoires differ depending on the disease [16]. A limitation of the current study is that samples were obtained from RA patients only. It is therefore possible that patients with other underlying diseases/infections may have RF with different functional properties. A further limitation is the lack of information regarding both the RF titre of each sample and the clinical features of the respective RA patients. It has been reported that the ability of monoclonal RF to induce complement deposition varies depending on the epitope recognised [16]. Therefore, the CDC activity of the RF repertoire present in sera may reflect the level of reactivity with a particular subset of epitopes rather than reactivity with all epitopes. This merits further investigation, particularly in the context of antigen-bound PD-1 antibodies which may have an optimal CDC epitope different to that observed in assays using solid-phase antibodies.

In this study, we provide the first demonstration that IgM-RF and IgA-RF bind cell surface bound PD-1/PD-L1 antibodies. The bound RF provided PD-1 antibodies with CDC activity but importantly did not modulate other Fc-associated functions associated with PD-1/PD-L1 antibodies. Additionally, bound RF provides a potential mechanism for signalling through IgA and IgM receptors [3]. The impact of RF on the Fc signalling and associated efficacy of therapeutic antibodies therefore merits further investigation.

## Supplementary Information

Below is the link to the electronic supplementary material.

Supplementary file1 (PDF 667 kb)

## Data Availability

The datasets generated during and/or analysed during the current study are available from the corresponding author on reasonable request.
