# Supplementary material for: Impact of rheumatoid factors on the function of therapeutic monoclonals specific for PD-1/PD-L1
Source: Cancer Immunol Immunother. 2025 May 24;74(7):216. doi: 10.1007/s00262-025-04078-0 (PMC12103427; doi:10.1007/s00262-025-04078-0)
Supplement: Supplementary file 1 — Supplementary file1 (PDF 667 kb) [file 262_2025_4078_MOESM1_ESM.pdf]

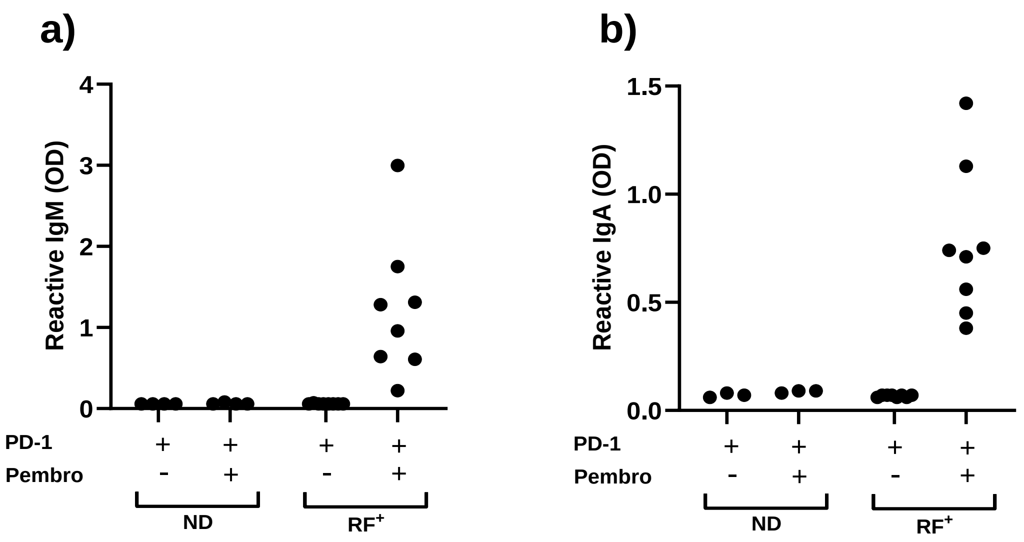

Supplementary Figure. 1 Reactivity of RF^+ and ND sera with solid phase PD-1 that had been pre-incubated with either nil or pembrolizumab. Binding was analysed by (a) IgM or (b) IgA specific ELISA and results from a representative experiment shown as a scatter plot of OD.

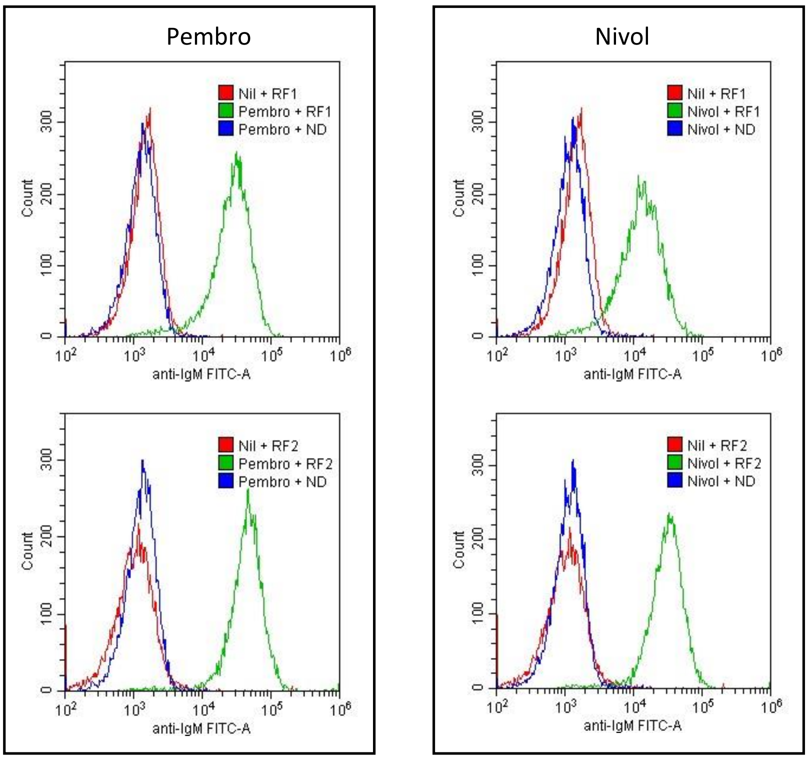

Supplementary Figure. 2 Comparison of IgM-RF reactivity with pembrolizumab or nivolumab bound to PD-1⁺ cells. PD-1⁺ cell line was labelled with either nil, nivolumab or pembrolizumab in combination with a ND or 2 individual RF⁺ sera. Bound IgM was detected by staining with Fitc-anti-IgM in combination with flow cytometry. Histograms of staining observed using the indicated combinations of antibodies are shown and are from a representative experiment of two performed.

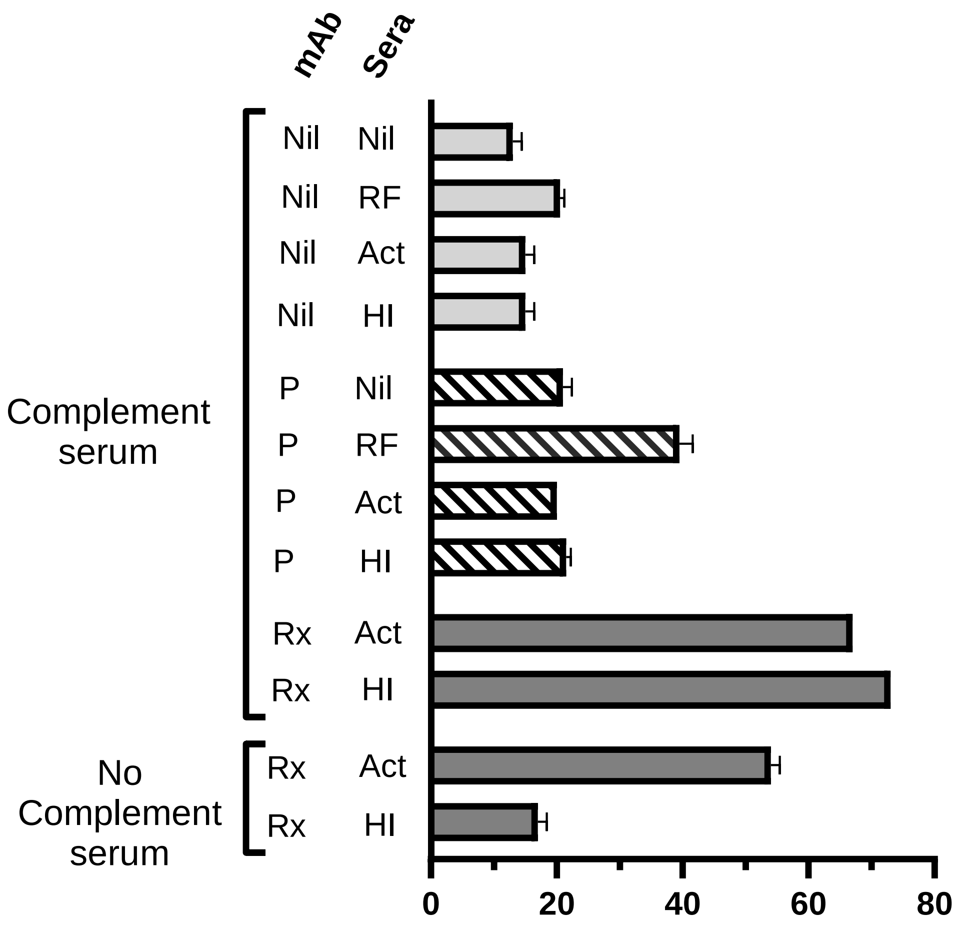

Supplementary Figure. 3 Effect on CDC of intrinsic complement activity within the test. PD-1⁺ Ramos was mixed with the indicated combinations of mAb (pembrolizumab (P), rituximab (Rx)) and test sera then cultured in the presence or absence of added complement serum. Test sera were either RF⁺ with established CDC inducing activity or healthy control serum that either had high complement activity (Act) or had been heat inactivated (HI). Data are shown as %PI⁺ (mean+ SEM) and are from a representative experiment of 2 performed. The samples containing rituximab plus Act or HI test sera but not further supplemented with complement sera acted as controls to demonstrate the complement activity of HI and Act test sera.

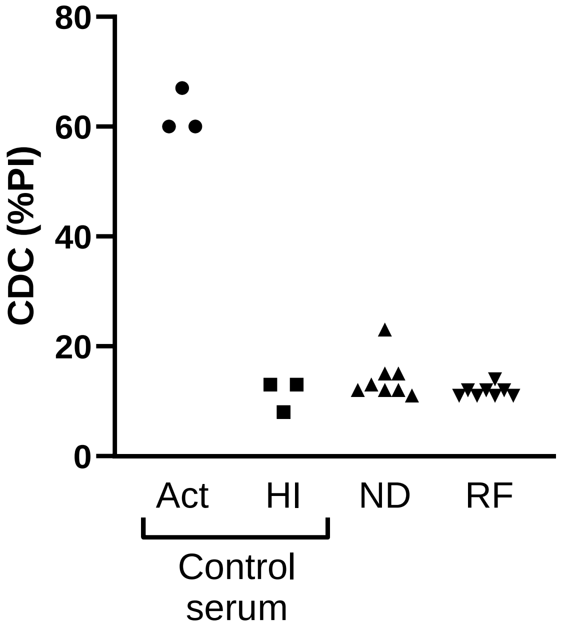

Supplementary Figure. 4 Complement activity in ND and RF^+ sera. PD-L1 Ramos was mixed with rituximab and the indicated combinations of test sera as in the normal CDC method. HBSS without additional complement serum was added and following 1h culture the % CDC determined. Sera analysed were either (i) Healthy control serum that had either full complement activity (Act) or had been heat inactivated (HI) (ii) ND (n=8) and RF^+ (n=8) test sera that had been used throughout the study.

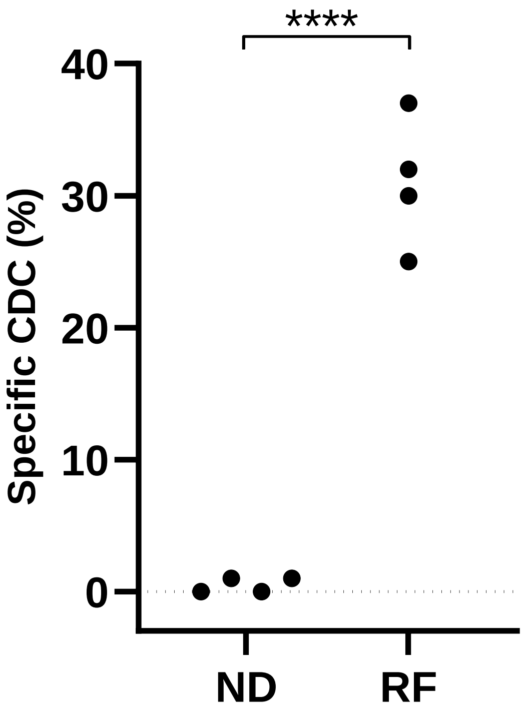

Supplementary Figure. 5 Effect on CDC of washing away test serum pre-complement addition. PD-1^{+} Ramos was incubated (30min) with either ND (n=4) or RF^{+} (n=4) serum in combination with either nil or pembrolizumab. Target cells were then washed prior to incubation in complement serum and determination of % specific CDC. Data are shown as a scatter plot and asterisks indicate statistical significance following comparison of ND and RF by t-test

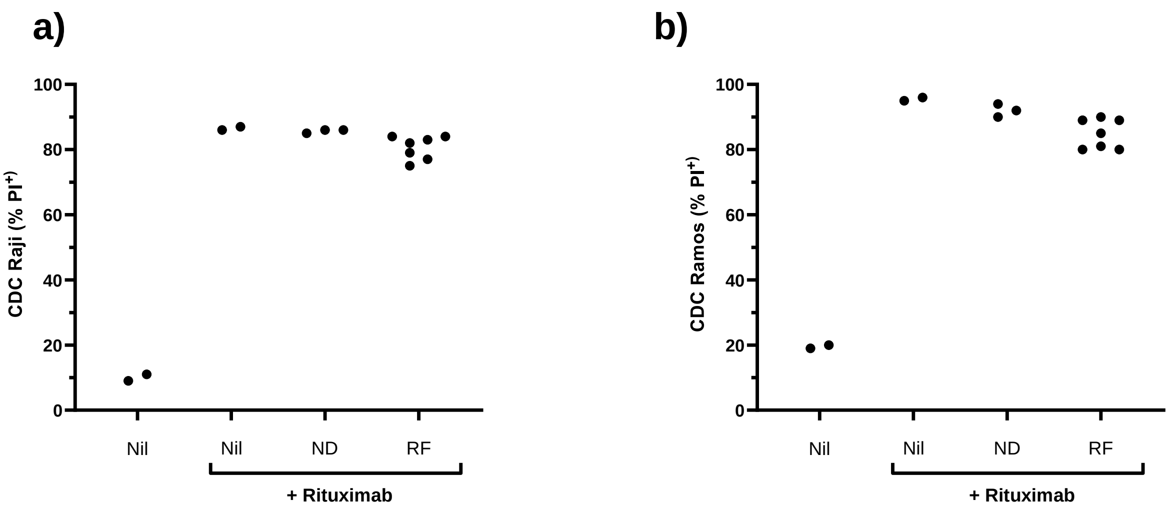

Supplementary Figure. 6 Effect of RF on rituximab induced CDC of B cell lymphoma lines. The cell lines (a) Raji and (b) Ramos were incubated with either media alone or rituximab in combination with nil, ND (n=3) or RF^+ (n=7) sera. Following exposure to human complement the % PI^+ was determined and data shown as a scatter plot of CDC observed with each individual serum. Data are from a representative experiment of 3 performed.

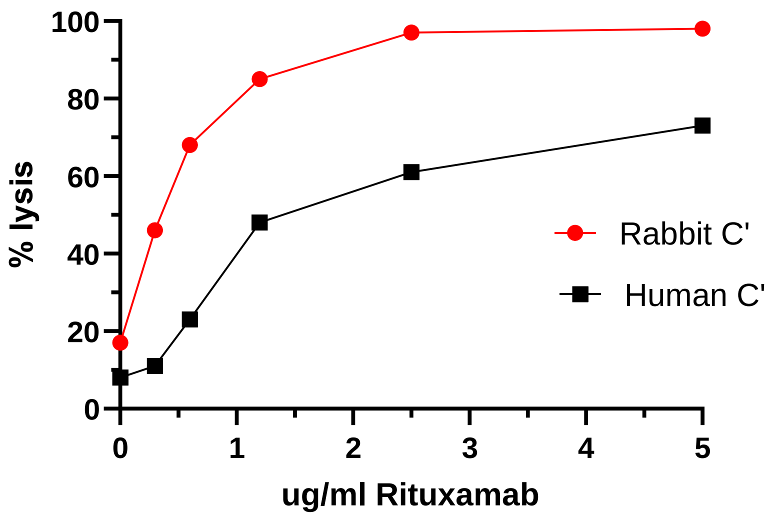

Supplementary Figure. 7

CDC of PD-1^+ Ramos that was incubated with the indicated concentration of rituximab then split and further incubated in the presence of 10% rabbit serum or 10% human serum as a complement source. Following 1h incubation, viability was accessed using PI staining and flow cytometry. Data are shown as a plot of CDC (%PI^+) versus rituximab concentration and are from a representative experiment of two performed.
